# Effects of Sleep Deprivation on Oral Health: A Literature Review

**DOI:** 10.7759/cureus.116949

**Published:** 2026-09-25

**Authors:** Sara Saba, Jad Sabbagh, Christelle Assaf, Pierre Khoury

**Affiliations:** 1 Faculty of Dentistry, Lebanese University, Beirut, LBN; 2 Prosthodontics, Saint Joseph University of Beirut, Beirut, LBN; 3 Prosthodontics, Lebanese University, Beirut, LBN

**Keywords:** obstructive sleep apnea, oral health, periodontal diseases, saliva, sleep deprivation

## Abstract

Oral health and sleep health are rarely discussed in the same clinical conversation, yet the biological evidence linking them is both substantial and growing. Chronic sleep deprivation triggers a cascade of systemic disruptions, including immune dysregulation, hormonal imbalance, and oxidative stress, which leave unmistakable marks on the oral cavity. Despite this, sleep analysis remains largely absent from routine dental assessment, and its role as an independent driver of oral disease has yet to receive the clinical attention it deserves. This article provides a comprehensive narrative review of the mechanisms through which sleep deprivation compromises oral health, organized around five interconnected domains: the immunological consequences of sleep loss, including its role in periodontal destruction and impaired mucosal healing; disruptions to the salivary environment and their downstream effects on caries, xerostomia, and halitosis; behavioral and lifestyle mediators such as glycemic dysregulation, tobacco use, and caffeine consumption; neuromuscular and structural consequences encompassing bruxism, temporomandibular dysfunction, traumatic dental injuries, and dysphagia; and obstructive sleep apnea as a major oral health comorbidity that both reflects and reinforces sleep deficiency. The article further addresses the practical implications of these findings for clinical dental practice, outlining how dentists can incorporate sleep-related screening into routine assessments through validated questionnaires, anatomical risk evaluation, and recognition of characteristic oral markers, and when referral to a sleep medicine specialist is warranted. The evidence reviewed here positions sleep deprivation as a significant, independent risk factor for a broad range of oral conditions and supports the integration of sleep health into a more comprehensive, patient-centered model of dental care.

## Introduction and background

Sleep is an essential biological process that plays a critical role in maintaining the health and well-being of individuals across all ages, genders, and backgrounds. Far beyond simple rest, sleep actively supports brain function and regulates numerous physiological systems throughout the body [1]. Both the duration and quality of sleep are key determinants of overall health outcomes, with guidelines from the American National Sleep Foundation recommending that healthy adults obtain between seven and nine hours of sleep per night, while children, adolescents, and older adults have their own distinct sleep requirements [2].

Scientific investment in sleep research has intensified in recent years in response to the widespread decline in sleep quality and duration across modern populations. This shift can be attributed to the adaptation to a 24/7 lifestyle culture, characterized by extended working hours, irregular shift schedules, and increasing professional and social demands that intrude on rest time. Medical conditions, household duties, and caregiving roles further restrict sleep in both adults and children, resulting in reduced sleep duration worldwide [1]. In addition, individual lifestyle choices play an obvious role in shaping sleep patterns. Factors such as prolonged screen exposure, heavy use of social media and the internet, psychological stress, dietary habits, smoking, and alcohol consumption have all been identified as contributors to disrupted or insufficient sleep. Collectively, these factors illustrate the complex nature of sleep health and emphasize the need to treat it as a significant public health priority [2].

Inadequate sleep has been associated with a broad range of adverse health outcomes. From a psychological standpoint, poor sleep has been linked to deteriorating mental health, manifesting as mood disturbances, memory impairment, heightened stress levels, diminished concentration, and overall declines in cognitive performance [3]. Beyond the brain, sleep plays a critical regulatory role across multiple physiological systems. When nightly sleep is chronically reduced, the consequences are far-reaching. Immune defenses weaken, systemic inflammation rises, hormonal balances are disrupted, and total daily energy expenditure increases. Sleep-deprived individuals are also more prone to adopting unhealthy dietary and lifestyle behaviors. Clinically, insufficient sleep is associated with an elevated risk of depression, obesity, hypertension, dyslipidemia, and type 2 diabetes. Sleep deprivation also contributes to cardiovascular disease and has been linked to premature mortality in epidemiological research. This suggests that consistent and restorative sleep is essential not only for daily functioning but also for strengthening the body's ability to fight infections and lowering the risk of disease [2,4].

The World Health Organization's landmark definition of health as "a complete state of physical, mental, and social well-being, not merely the absence of disease" (WHO, 1948) laid the groundwork for understanding health as a multidimensional construct. Oral health, in this context, extends well beyond the absence of dental disease. It encompasses an individual's capacity to eat, speak, and express emotions comfortably and confidently, while also influencing self-esteem, social interaction, academic performance, and professional life. Achieving good oral health implies that an individual is not burdened by chronic pain, active infections, or debilitating conditions that can severely undermine the overall health of the oral cavity [2].

Research has established links between certain oral and dental conditions, including dentognathic deformities (structural abnormalities of the teeth and jaws), tooth loss, and periodontal disease, and the development of sleep disturbances such as obstructive sleep apnea (OSA) and compromised sleep quality. However, the reverse relationship remains largely unexplored, as little attention has been given to understanding whether poor sleep health itself may contribute to deteriorating oral health and its impact on quality of life [5,6].

These systemic disruptions do not remain confined to the rest of the body. The same inflammatory, hormonal, and oxidative pathways triggered by sleep loss extend into the oral cavity, where they compromise periodontal tissue, salivary function, and the neuromuscular control of the jaw and pharynx [2,4,7]. Periodontal disease, the chronic inflammatory destruction of the gingiva, periodontal ligament, and alveolar bone that support the teeth, offers one of the clearest illustrations of this overlap, as the pro-inflammatory cytokines elevated by insufficient sleep are the same mediators implicated in periodontal tissue breakdown [2,7]. This review traces the overlap across five interconnected domains: the immunological consequences of sleep loss on periodontal and mucosal tissue; disruptions to the salivary environment and their effects on caries, xerostomia, and halitosis; behavioral and lifestyle mediators such as glycemic dysregulation, tobacco use, and caffeine consumption; neuromuscular and structural consequences including bruxism, temporomandibular dysfunction, traumatic dental injuries (TDIs), and dysphagia; and obstructive sleep apnea as a condition that both reflects and reinforces sleep deficiency.

This article aims to provide a comprehensive narrative review of the mechanisms through which sleep deprivation compromises oral health, organized around five interconnected domains: the immunological consequences of sleep loss, disruptions to the salivary environment, behavioral and lifestyle mediators, neuromuscular and structural consequences, and obstructive sleep apnea as a major oral health comorbidity. In doing so, it also addresses the practical implications of these findings for clinical dental practice, outlining how dentists can incorporate sleep-related screening into routine assessments and when referral to a sleep medicine specialist is warranted.

## Review

Methods

A comprehensive narrative literature review was conducted involving a systematic search for relevant articles across PubMed/Medical Literature Analysis and Retrieval System Online (MEDLINE), Scopus, and Google Scholar databases. The search utilized a combination of Medical Subject Headings (MeSH) terms and high-precision free-text keywords structured via Boolean operators (AND/OR), including "sleep deprivation," "sleep quality," "oral health," "periodontal disease," "dental caries," "xerostomia," "halitosis," "bruxism," "temporomandibular disorders," "dysphagia," "obstructive sleep apnea," "salivary flow," "systemic inflammation," "glucose metabolism," "caffeine and sleep," and "tobacco use and sleep." The period considered for study selection ranged from 2000 to 2026. Inclusion criteria consisted of clinical trials, observational studies, cross-sectional studies, systematic reviews, meta-analyses, and narrative reviews published in English that specifically addressed the relationship between sleep deprivation or sleep quality and oral or systemic health outcomes. Studies were eligible if they reported physiological, biochemical, behavioral, or clinical findings relevant to the oral cavity in the context of insufficient or disrupted sleep. Exclusion criteria included studies focusing exclusively on sleep architecture without reporting oral or systemic health outcomes, purely theoretical or opinion-based papers lacking empirical support, non-peer-reviewed sources, and articles published in languages other than English. The initial search results were screened by two independent reviewers to minimize selection bias, with any discrepancies resolved through consensus. After the removal of duplicates and application of inclusion and exclusion criteria, a total of 58 articles were included in this review. Due to the considerable heterogeneity in study designs, populations, and outcome measures across the included literature, a quantitative meta-analysis was not performed. Instead, a narrative synthesis was conducted, following principles consistent with the spirit of the Preferred Reporting Items for Systematic Reviews and Meta-Analyses (PRISMA) guidelines for transparency, to allow a comprehensive and flexible exploration of the multidimensional relationship between sleep deprivation and oral health. As a narrative rather than a systematic review, this approach was intended to enhance transparency of reporting rather than to fulfill the full methodological requirements of a systematic review, such as a PRISMA flow diagram, per-database Boolean search strings, or formal risk-of-bias assessment of individual studies; these considerations are discussed further in the Limitations section. The findings were organized thematically across five interconnected domains: immunological consequences, salivary environment disruptions, behavioral and lifestyle mediators, neuromuscular and structural consequences, and obstructive sleep apnea as a major oral health comorbidity.

Immunological consequences of sleep deprivation

Sleep Deprivation and Periodontitis

As introduced, the immune and inflammatory dysregulation triggered by poor sleep extends its consequences directly to the oral cavity, creating a destructive feedback loop with existing oral pathologies [2]. Both sleep deprivation and periodontal disease are fundamentally characterized by amplified, low-grade chronic inflammatory responses within the body. This overlapping presentation forms a shared biological pathway that identifies inadequate sleep as a significant, independent risk factor for oral tissue destruction [2,4].

When an individual experiences sleep deprivation, whether acute or chronic, the body's natural immune mechanisms are compromised, and endocrine balance is disrupted [2]. This systemic stress alters normal cellular responses, leading to an overproduction and daytime shift of critical pro-inflammatory cytokines and signaling molecules, most notably interleukin-1 (IL-1), interleukin-6 (IL-6), interleukin-17 (IL-17), and tumor necrosis factor-alpha (TNF-α). These are the exact same inflammatory mediators that drive the pathogenesis and cellular progression of periodontal disease [2,7]. Because these specific cytokines play a central role in breaking down structural collagen and causing periodontal tissue destruction, their elevated systemic levels in sleep-deprived individuals directly potentiate the clinical severity and rapid progression of the disease [2].

This proposed biological mechanism is strongly reinforced by clinical and epidemiological findings across multiple adult populations. Individuals with advanced periodontal conditions consistently demonstrate poorer overall sleep quality and shorter sleep durations [2,7]. Clinical measurements reveal that lower sleep scores correlate positively with greater pocket probing depths, advanced clinical attachment loss, and heightened differences in white blood cell markers [8]. Ultimately, the relationship between oral pathologies and sleep restriction highlights how systemic inflammatory markers, including IL-6 and white blood cell levels, interact across chronic conditions [7]. This suggests that integrating sleep evaluation into routine dental assessments could provide clinicians with a more comprehensive understanding of a patient's underlying risk factors for periodontal progression [8].

A pilot study by Grover et al. found a clear clinical association between sleep deprivation and periodontal disease severity, with patients with moderate-to-severe periodontitis showing significantly poorer sleep quality than those with gingivitis or healthy controls. This link may stem from sleep restriction's activation of the sympathetic nervous system (SNS), which promotes systemic inflammation and can accelerate periodontal tissue destruction [1]. The literature also indicates a U-shaped relationship between sleep duration and periodontal risk, where both insufficient and excessive sleep are associated with a higher likelihood of periodontitis (Figure 1) [7].

Nevertheless, the bidirectional nature of this link suggests that while sleep loss drives inflammation, inflammatory cytokines also play a role in the homeostatic regulation of sleep, creating a cycle where targeting one may benefit the other [8].

**Figure 1:**
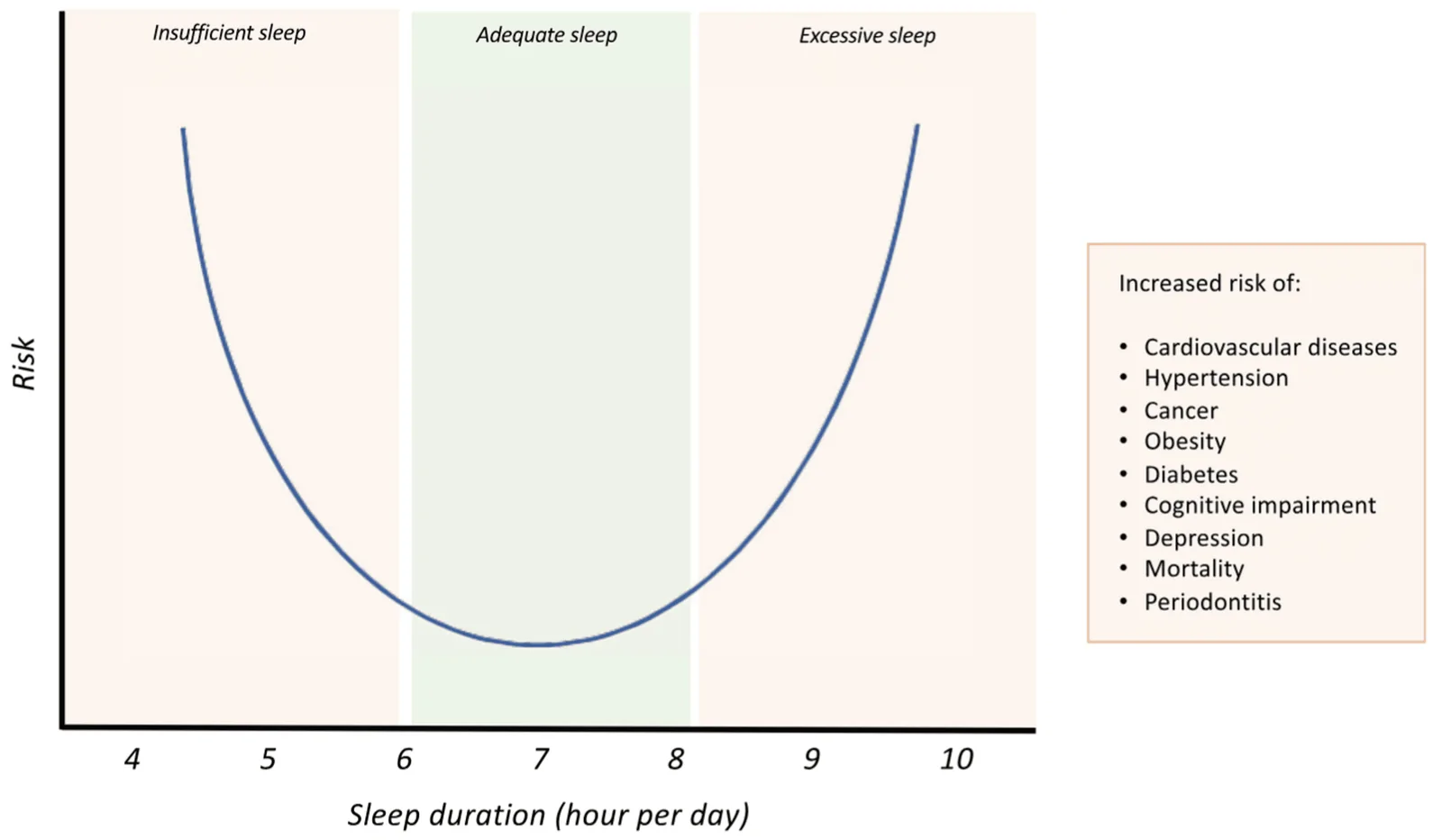
A U-shaped model representing how daily sleep duration impacts disease risk. The trajectory demonstrates that both inadequate rest (generally <6 hours per day) and excessive sleep (generally >8 hours per day) correlate with a heightened risk for multiple pathologies, such as periodontal disease. Figure reproduced from Carra et al. [7], licensed under a Creative Commons Attribution 4.0 International License (CC BY 4.0) (https://creativecommons.org/licenses/by/4.0/).

Impaired Mucosal Healing and Oral Ulceration

As a frequent condition affecting the oral mucosa, mouth ulcers can significantly diminish a patient's overall well-being and daily functioning [9].

The etiology of these lesions is multifactorial; they may be triggered by physical injury, recurrent aphthous stomatitis (RAS), or diverse microbial infections. Furthermore, mucosal ulcerations may stem from allergic responses, adverse drug interactions, or underlying systemic health issues [10]. While the exact pathogenesis remains a subject of ongoing research, the susceptibility to developing these sores is influenced by a combination of genetic predisposition, sedentary lifestyle, and suboptimal oral hygiene [11].

Recent research suggests that sleep quality is a pivotal, yet underappreciated, factor in this equation. A study by Chen et al. in experimental rat models showed that sleep deprivation significantly worsens inflammation and slows the healing of existing ulcers. These findings highlight a clear correlation between insufficient rest and increased ulcer dimensions, alongside a marked decline in regenerative efficacy compared to subjects with normal sleep cycles [9].

This correlation is driven by several distinct physiological shifts. When sleep is restricted, the body undergoes a significant surge in the same pro-inflammatory cytokine family discussed in the Sleep Deprivation and Periodontitis section, along with IL-8; these molecular markers promote edema and exacerbate the destruction of oral mucosal tissues [9]. Lack of rest also leaves oxidative stress unchecked, marked by elevated malondialdehyde (MDA) and 8-OHdG levels and delayed recovery of protective superoxide dismutase (SOD) enzymes, an imbalance that further hinders the healing of mucosal sores [12,13]. Neurotransmitter changes add another layer of complexity: sleep loss has been linked to increased serotonin and gamma-aminobutyric acid (GABA) levels in serum and brain tissue. While GABA is normally protective, its benefits appear to be outweighed by the pro-inflammatory state caused by total sleep deprivation [9]. These biological observations are supported by human epidemiological data. For instance, Liu et al. found that longer sleep durations are significantly associated with a reduced risk of mouth ulcers, whereas insomnia correlates with an elevated risk profile [14].

Ultimately, maintaining adequate sleep is essential for preserving immune system balance and protecting the oral mucosa from chronic ulceration [9].

The salivary environment and chemical shifts

Salivary Hypofunction and Xerostomia

Often overlooked in daily life, saliva is one of the oral cavity's most essential defenders. Far more than a simple lubricant, it performs a remarkable range of functions that are critical to maintaining oral health: it mechanically rinses away food debris and bacteria, neutralizes acidic challenges to tooth enamel through its buffering capacity, delivers antimicrobial proteins such as lysozyme and immunoglobulin A to the mucosal surfaces, and facilitates the very first steps of digestion. In short, a healthy salivary flow is what keeps the oral environment chemically balanced and microbiologically under control [15,16]. When that flow diminishes (a condition clinically referred to as hyposalivation or subjectively experienced as xerostomia (the feeling of oral dryness)), the consequences ripple across virtually every aspect of oral health. Without the constant washing and buffering action of saliva, pathogenic bacteria are left to proliferate unchecked, volatile sulfur compounds accumulate and give rise to halitosis, and dietary acids are no longer neutralized efficiently, leaving enamel surfaces vulnerable to demineralization and eventually caries. The mouth, stripped of its primary chemical shield, becomes a far more hostile environment [16].

What makes this particularly relevant to the discussion of sleep is that salivary production is not static. It follows a well-established circadian rhythm, naturally declining during the nighttime hours [17]. This physiological reduction is normal and generally manageable in healthy individuals who sleep well. However, when sleep itself is compromised, this already-reduced nocturnal flow can deteriorate further. A cross-sectional clinical study by Apessos et al. investigating the relationship between sleep disorders and xerostomia found that individuals with objectively measured morning hyposalivation, assessed using the oral modified Schirmer test, exhibited significantly poorer sleep quality compared to those with normal salivary flow (hyposalivation group n=63, control group n=110; p < 0.001), as measured by the Pittsburgh Sleep Quality Index (PSQI). Notably, over two-thirds of subjects in the hyposalivation group were classified as poor sleepers, compared to just over one-third in the control group (Figure 2) [16]. The structure and architecture of sleep in patients with dry mouth appear to be disturbed, and this disturbance has been associated with poor sleep quality and excessive daytime sleepiness [18]. Moreover, the relationship seems to operate in a self-reinforcing loop: nocturnal xerostomia leads to frequent awakenings to drink water, which in turn triggers urination awakenings, creating a vicious cycle that makes returning to sleep increasingly difficult. The mouth, in this sense, becomes both a victim and a signal of disrupted sleep, and the downstream consequences for dental health are far from trivial [16].

**Figure 2:**
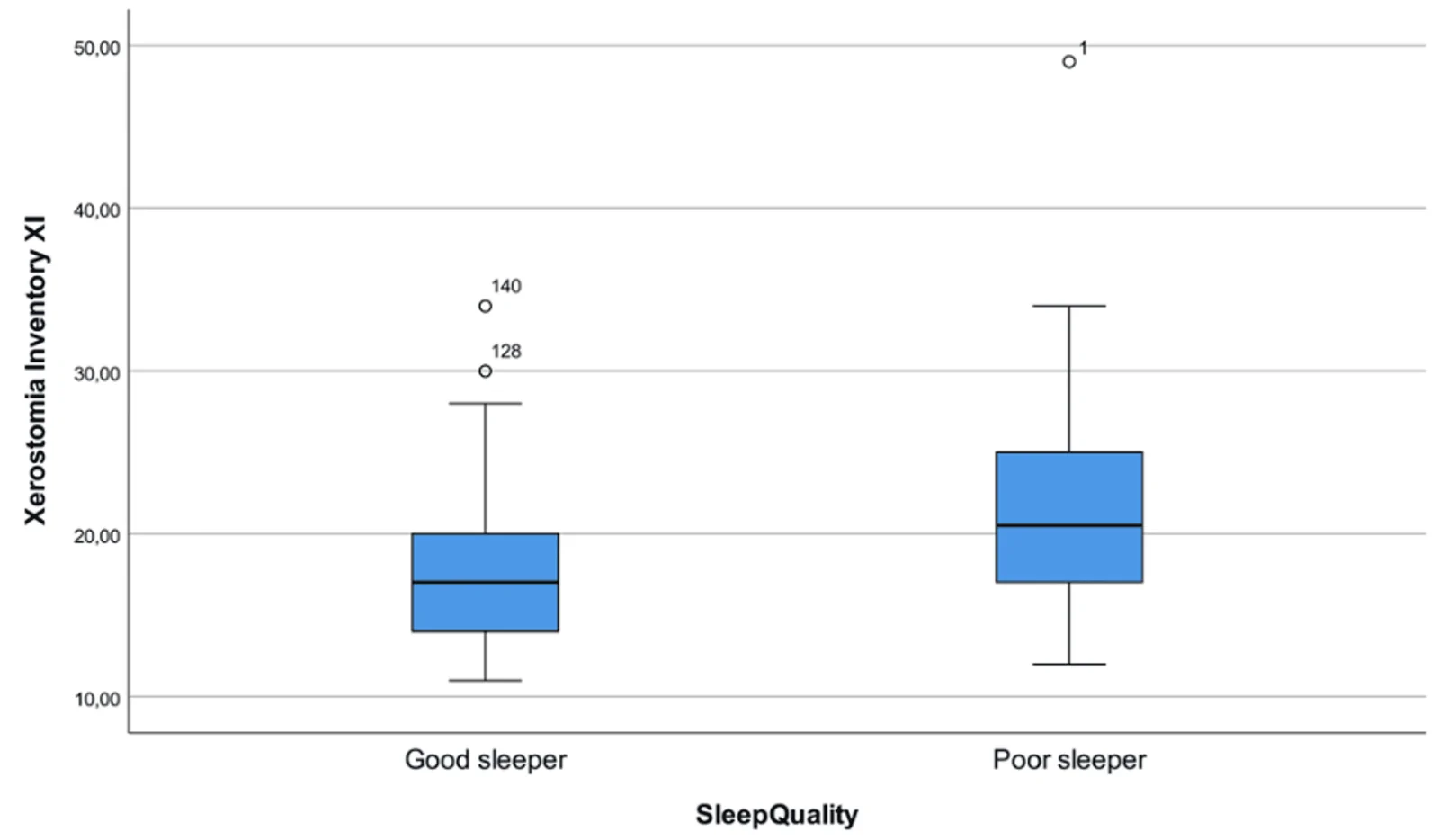
Box plot of XI levels per subgroups of PSQI. Higher values of XI were correlated with poor sleep quality (p < 0.001) PSQI: Pittsburgh Sleep Quality Index. Figure reproduced from Apessos et al. [16], licensed under a Creative Commons Attribution 4.0 International License (CC BY 4.0) (https://creativecommons.org/licenses/by/4.0/).

Dental Caries and Enamel Vulnerability

Those consequences begin with caries. Saliva's buffering capacity depends on its production at rest, and a reduction in nighttime flow leaves enamel surfaces exposed to the acidic byproducts of bacterial metabolism for longer, uninterrupted periods. Cariogenic bacteria such as *Streptococcus mutans* thrive in this environment, and research has confirmed that xerostomic patients harbor elevated counts of these acidogenic species [19]. Experimentally, Lasisi et al. demonstrated in a controlled animal study that total sleep deprivation over seven days was associated with a significant reduction in salivary flow rate and a concurrent drop in secretory IgA secretion rate, the antibody that forms the primary mucosal defense against bacterial colonization. Without adequate IgA delivery, pathogenic bacteria encounter reduced resistance, accelerating plaque formation and enamel demineralization. In addition, reduced salivary flow directly diminishes the oral cavity's cleaning and diluting capacity, allowing fermentable carbohydrates and bacterial acids to linger on tooth surfaces much longer than normal [15]. Beyond mechanical clearance, hyposalivation also depletes the protective proteins and electrolytes responsible for neutralizing bacterial acids and suppressing cariogenic microorganisms, a dual loss that accelerates the caries process considerably [19].

Halitosis and Oral Bacterial Proliferation

An equally immediate but often underappreciated consequence of this nocturnal salivary reduction is the promotion of bad breath. Halitosis is multi-causal in origin, arising from oral-related factors such as periodontitis, poor oral hygiene, tongue coating, and salivary gland dysfunction, as well as systemic conditions including leukemia, diabetic ketoacidosis, respiratory disorders, stomatitis, and medication use [20-22]. Yet among these varied triggers, the absence of adequate salivary flow during sleep stands out as one of the most physiologically direct and consistently documented [20]. During sleep, salivary flow drops dramatically, and when this is further compounded by poor or insufficient sleep, the oral environment shifts in ways that actively favor malodor. The reduced flow allows retained exfoliated oral epithelial cells and food debris to undergo bacterial degradation. This reaction generates volatile sulfur compounds, the primary chemical agents responsible for bad breath, in a process significantly amplified when sleep deprivation suppresses salivary output beyond the normal nocturnal decline [20,23]. The drop in IgA secretion documented by Lasisi et al. is particularly relevant here, as it removes the oral cavity's first immunological line of defense, allowing bacterial colonization on teeth and tongue to accelerate and halitosis to intensify as a direct result [15]. Resting salivary flow rate has, in fact, been identified as an independent explanatory factor in clinical findings of oral malodor, reinforcing that the volume and quality of saliva, and not hygiene habits alone, determine the severity of bad breath [24].

Sleep deprivation also drives halitosis through a behavioral pathway that compounds its biological effects. The mental fatigue and heightened stress generated by insufficient sleep frequently lead to irregular oral hygiene practices such as skipping tooth brushing, which in turn drives plaque buildup on both teeth and the tongue. In a large cross-sectional study of over 62,000 Korean adolescents, Do (2020) found that those who reported their sleep to be "not at all sufficient" were more than twice as likely to experience bad breath compared to those who reported completely sufficient sleep, with the association remaining statistically significant even after adjusting for all confounding variables including smoking, stress, and oral hygiene frequency. Sleep is therefore more than just rest; its disruption triggers xerostomia, which in turn creates the oral conditions for both caries and bad breath to develop [24].

Behavioral and lifestyle mediators

Glycemic Dysregulation and Dietary Shifts

Sleep is integral to a range of physiological processes, including glucose metabolism, appetite regulation, endocrine function, and immune response [25]. When sleep is chronically insufficient, the consequences extend well beyond fatigue, contributing to conditions such as obesity, insulin resistance, type 2 diabetes, metabolic syndrome, and cardiovascular disease [26]. Notably, even a single night of poor sleep can produce immediate cognitive, physical, and metabolic disruptions, and this applies not only to individuals with pre-existing metabolic conditions, but to otherwise healthy, non-diabetic people as well [25,26].

Sleep deprivation destabilizes glucose homeostasis through both direct endocrine pathways and oblique behavioral mechanisms. Biologically, sleep loss triggers systemic metabolic dysregulation via several concurrent pathways, primarily involving sympathetic nervous system overstimulation, which directly interferes with insulin signaling. This autonomic overactivation is compounded by marked shifts in appetite-regulating hormones, specifically leptin, ghrelin, and peptide YY (PYY), alongside elevations in circulating glucose, insulin, glucocorticoids, catecholamines, free fatty acids, and triglycerides. Furthermore, this state induces subclinical inflammation characterized by elevated levels of pro-inflammatory cytokines [25,26]. Consequently, in healthy individuals, sleep loss acutely diminishes peripheral insulin sensitivity, and because pancreatic beta-cell function fails to upregulate and compensate for this resistance, temporary impaired glucose tolerance develops [26].

This biochemical disruption directly drives the "Exhaustion Cycle" at a behavioral level, significantly altering total caloric consumption and generating intense cravings for highly sweet or salty foods [26]. Quantifying this effect, a systematic review and meta-analysis by Zhu et al., conducted under controlled laboratory conditions, found across 41 randomized controlled trials that partial sleep restriction was associated with an increase of approximately 253 kcal per day compared with normal sleep duration (p=0.011) [27]. Similarly, a systematic review and meta-analysis by Al Khatib et al. examining both laboratory and free-living settings reported that sleeping five and a half hours or less led to a pooled increase of 385 kcal per day (95% CI: 252-517; 11 studies, n=172), a higher proportion of fat intake, and a reduction in protein consumption [28].

From a neurological perspective, sleep-deprived individuals exhibit reduced activity in brain regions governing food choice, theoretically predisposing them to poorer dietary decisions. They simultaneously show heightened activity in regions that signal food reward value and drive eating behavior. Additionally, evidence suggests that sleep-deprived individuals consume more dietary fat, or an excess of both fat and carbohydrates combined [29]. This chronic elevation in circulating and salivary glucose, combined with increased intake of fermentable carbohydrates, provides a more favorable substrate for acid-producing oral bacteria, linking sleep-driven glycemic dysregulation directly to an elevated risk of dental caries [26,28].

Sleep Deprivation and Tobacco Use

The relationship between sleep and tobacco use is bidirectional and well-documented. Compared to non-smokers, smokers are more likely to experience inadequate sleep duration, and nighttime smoking has a direct effect of fragmenting and shortening sleep in 41% of cases [30,31].

This relationship becomes particularly problematic when a smoker attempts to quit. During cessation, the body goes through nicotine withdrawal, and insomnia is one of its clinically recognized symptoms. In other words, quitting smoking can itself disrupt sleep, which in turn increases irritability, impairs concentration, and raises the likelihood of relapse [32]. Adding further complexity, some of the most commonly prescribed cessation medications, such as varenicline and transdermal nicotine patches, can also cause or worsen sleep disturbances as a side effect, creating a situation where the treatment itself may undermine recovery [31,32].

What makes this relationship particularly concerning is its self-reinforcing nature. A laboratory study by Hamidovic and De Wit demonstrated this directly by testing abstinent smokers after a night of total sleep deprivation and after a night of normal sleep. Participants were given repeated choices between smoking a cigarette or receiving a small cash reward. After sleep deprivation, smokers consistently chose cigarettes over money more often than they did when well-rested. This suggests that sleep loss increases the perceived need for a cigarette, likely because smokers learn from experience that nicotine can temporarily relieve fatigue and restore concentration. Importantly, increased smoking after sleep deprivation was positively correlated with craving scores on the stimulation-seeking subscale, reinforcing the idea that tired individuals reach for cigarettes as a functional coping mechanism rather than as a purely habitual response [33].

At the population level, a study by Patterson et al. showed that smokers transitioning from adequate (seven to eight hours) to inadequate sleep duration were more likely to smoke more cigarettes per day, to find it difficult to go without smoking for a day, and to develop higher nicotine dependence. However, the reverse transition, from inadequate to adequate sleep, did not produce a corresponding reduction in smoking behaviors, suggesting that once sleep deprivation accelerates nicotine dependence, the habit becomes increasingly difficult to break [30].

Caffeine, Sleep, and the Compounding Effect on Health

Caffeine is the most widely consumed psychoactive stimulant in the world, with approximately 80% of the global population ingesting it regularly, most commonly through coffee, tea, and energy drinks. Its appeal is rooted in its ability to promote wakefulness and temporarily counteract the cognitive and physical consequences of fatigue. Pharmacologically, caffeine works by blocking adenosine receptors in the brain, the same receptors targeted by adenosine, a compound that accumulates during wakefulness and progressively signals the need for sleep [34]. By occupying these receptors, caffeine suppresses the perception of sleepiness and sustains alertness. This mechanism is precisely why sleep-deprived individuals reach for caffeine as a coping strategy, making it the most socially normalized response to insufficient sleep [35].

The problem, however, is that caffeine does not simply mask tiredness without consequence. It actively disrupts the sleep that follows. A systematic review and meta-analysis by Gardiner et al. showed that caffeine reduces total sleep time by delaying sleep onset and lowering overall sleep efficiency. Beyond duration, caffeine also alters the structure of sleep itself, increasing the proportion of light sleep and reducing deep sleep, the specific physiological window required for cellular repair, tissue regeneration, and immune function. The same meta-analysis established that a standard cup of coffee must be consumed at least 8.8 hours before bedtime to avoid a statistically significant reduction in total sleep time, meaning that an afternoon coffee taken at 2 PM can still meaningfully impair a 10 PM sleep onset [35].

When caffeine intake occurs concurrently with acute or chronic sleep restriction, the physiological damage extends beyond simple neuro-cortical arousal. A randomized crossover clinical trial by Rasaei et al. investigated the simultaneous effects of sleep deprivation and coffee intake on healthy individuals. It demonstrated that in acutely sleep-deprived individuals, coffee consumption negatively affects glucose homeostasis, leading to elevated post-oral glucose tolerance test (OGTT) glucose and insulin levels, higher fasting insulin, and increased insulin resistance compared to decaffeinated controls [36]. From a dental standpoint, this temporary state of insulin resistance and high insulin levels raises glucose availability in both the bloodstream and saliva. In the mouth, this extra glucose feeds acid-producing bacteria, pushing the oral biofilm toward a more cariogenic profile and speeding up enamel demineralization [36,37].

Furthermore, the combined stress of sleep restriction and caffeine consumption triggers an unfavorable immuno-inflammatory and cardiovascular response. Mateo et al. found that acute caffeine intake during prolonged sleep deprivation worsened the hypertensive response and raised circulating IL-6, one of the same inflammatory markers discussed in the Sleep Deprivation and Periodontitis section, with chronic habitual consumption showing similar effects on blood pressure and IL-6 levels [7,37,38].

For the dental practitioner, these findings carry clinical relevance. Caffeine-driven IL-6 elevation during sleep loss fuels the inflammatory cascade behind periodontal tissue destruction, weakening gingival defenses. Additionally, the rise in blood pressure and autonomic changes suppress salivary flow, inducing xerostomia. Consequently, the sleep-deprived patient who relies on caffeine is caught in a destructive cycle: reduced salivary buffering, elevated glucose feeding acid-producing bacteria, and a systemic inflammatory surge accelerating periodontal breakdown [7,15,16,37].

Neuromuscular and structural consequences

Neurocognitive Impairment and Physical Vulnerability

Sleep deprivation triggers a cascade of neurocognitive dysfunctions that exceed simple fatigue. At the neurological level, prolonged sleep loss primarily disrupts prefrontal cortex functioning, impairing critical faculties such as attention, working memory, decision-making, and response inhibition. These cognitive impairments are mediated through decreased alertness, slowed processing speeds, and wake-state instability, which manifest as attentional lapses caused by brief microsleeps [3].

Crucially, the behavioral manifestation of this neurological strain varies by age. Rather than inducing the excessive daytime drowsiness typically seen in adults, insufficient sleep in younger individuals paradoxically manifests as hyperactivity, increased impulsivity, mood changes, oppositional behaviors, and attention deficits. These neurobehavioral alterations have direct physical consequences, leading to degraded motor coordination and compromised self-regulatory capacity [39,40].

The burden of sleep deprivation, however, is not purely cognitive. It carries a significant emotional toll as well. Poor sleep quality is consistently associated with elevated negative affect and diminished positive emotions, with substantial evidence linking even short-term sleep loss to measurable increases in anxiety and psychological distress [41]. The impact is severe enough that prolonged sleep deprivation has been classified as a form of torture in imprisonment contexts and is explicitly prohibited under international human rights frameworks [26]. A single night of inadequate sleep negatively impacts positive emotional states [26,41].

These combined neurobehavioral and emotional effects increase the risk of falls and unintentional collisions in sleep-deprived individuals [26,39-41]. As detailed below, this elevated risk of falls and collisions translates directly into a greater likelihood of orofacial and dental trauma, particularly among children.

Traumatic Dental Injuries

It is through these neurocognitive and motor deficits that sleep deprivation becomes highly relevant to clinical oral health, specifically regarding traumatic dental injuries (TDIs). TDIs represent a major public health concern, carrying a global prevalence of approximately 18.1% among 12-year-old children, and their etiology involves a complex network of biological, anatomical, and environmental risk factors [39]. Because accidental falls are the primary mechanism through which anterior dental trauma occurs in children, the clumsiness, inattention, and impulsivity generated by inadequate sleep create a highly plausible pathway toward oral trauma [39,40].

Daytime naps offer a vital protective mechanism against accidental injuries in younger children. Skipping a daytime nap is associated with a twofold increase in the risk of accidental falls, emphasizing the importance of preserving daytime rest periods to reduce fatigue-induced physical errors [40].

Bruxism and Temporomandibular Dysfunction

Sleep bruxism (SB) is defined as a masticatory muscle activity occurring during sleep, characterized by rhythmic (phasic) or non-rhythmic (tonic) patterns, and is not considered a movement or sleep disorder in otherwise healthy individuals [42-44]. Both sleep bruxism and its daytime counterpart, awake bruxism, are now understood to be behaviors that can be harmless, harmful, or even protective, depending on their clinical effects. Among the negative sequelae that may arise are mechanical tooth wear, crown and root fractures, restorative and prosthetic failures, and pain in the masticatory muscles and temporomandibular joints [44].

Evidence increasingly points to a relationship between sleep quality and bruxism, though the nature of this association varies depending on how each is measured. In a cross-sectional study of school-aged children, poor sleep quality was significantly associated with a prevalence of SB nearly 80% higher than in children who slept well, particularly in the 8-10 age group [43]. After a night of total sleep deprivation, healthy adults showed a significant drop in SB events during the first night of recovery sleep. This is thought to result from a rebound increase in deep slow-wave (N3) sleep, which temporarily restored normal sleep architecture and lowered stress levels. This observation aligns with the suggestion that improving sleep quality, and particularly attenuating the psychological stress that accompanies poor sleep, may reduce the frequency of bruxism episodes [42].

Disrupted sleep carries significant consequences for the temporomandibular joint itself. In a large prospective cohort, sleep quality declined over time only in initially temporomandibular disorders (TMDs)-free adults who later developed painful TMD, while it stayed stable in those who remained TMD-free [45]. Individuals with poor baseline sleep quality were twice as likely to develop first-onset TMD compared to good sleepers. Even after accounting for other contributing factors like psychological stress, somatic awareness, and comorbid health conditions, their risk of developing TMD stayed 73% higher. These findings serve as a reminder that sleep disturbance, if left unaddressed, may silently pave the way for the development of painful orofacial conditions [45].

Dysphagia and Oropharyngeal Motor Impairment

Sleep deprivation also compromises the neuromuscular mechanisms that govern swallowing. The act of swallowing is a precisely coordinated sequence requiring intact nerve signaling and muscle responsiveness, and both are vulnerable to sleep-related dysfunction. During sleep, pharyngeal swallowing pressure is naturally reduced in healthy adults, increasing the likelihood of accidental aspiration, and this risk is further amplified when swallowing function is already compromised [46].

Hama et al. found that among 3,058 older adults, those at risk for dysphagia were nearly twice as likely to report poor sleep quality, with shorter sleep duration and more irregular sleep patterns [46]. Dysphagia risk affected multiple aspects of sleep in both sexes, including sleep quality, time to fall asleep, nighttime disturbances, and daytime functioning. This points to a bidirectional relationship in which impaired swallowing disrupts sleep through mechanisms like nocturnal aspiration and reflux, while poor sleep further weakens the neuromuscular coordination swallowing depends on [47,48].

Daytime dysfunction, a direct consequence of fragmented or insufficient sleep, was associated with roughly twice the likelihood of swallowing difficulties, suggesting that the neurological toll of sleep deprivation does not spare the oropharyngeal system [46,49]. In addition, reduced saliva production during sleep also weakens the throat and esophagus's ability to clear and buffer acid, making nighttime reflux more likely. This triggers coughing, which disrupts sleep further and worsens swallowing problems, creating a self-perpetuating cycle [46]. From an oral health standpoint, this matters because dysphagia alters the oral environment: food residue clearance is delayed, mucosal contact with acidic or fermentable material is prolonged, and the protective mechanical action of swallowing is reduced, all of which increase bacterial load and the risk of caries, mucosal irritation, and aspiration-related complications [50].

Obstructive sleep apnea as a major oral health comorbidity

The relationship between sleep and oral health reaches its most clinically significant expression in obstructive sleep apnea (OSA), a condition affecting between 9% and 38% of the general population and representing the most common form of sleep-disordered breathing. OSA is defined by repeated episodes of partial or complete upper airway collapse during sleep, leading to oxygen desaturation, fragmented sleep architecture, and chronic sleep deficiency, all of which create a cascading burden on systemic and oral health [51].

*Obstructive Sleep Apnea* *(OSA) and Sleep Deprivation: A Bidirectional Relationship*

Critically, OSA and sleep deprivation may reinforce each other: sleep loss weakens the upper airway dilator muscles that keep the upper airway open, increases rapid eye movement (REM) sleep (when apneic events are more frequent), and blunts the body's response to low oxygen, all of which worsen airway obstruction. In patients with OSA, this means that inadequate sleep does not offer recovery; instead, it feeds back into the disorder itself, deepening the cycle of airway collapse, oxygen desaturation, and further sleep fragmentation [52]. The swallowing reflex, already discussed as a target of sleep deprivation, is specifically and measurably impaired in OSA patients who show delayed swallowing initiation, shortened post-swallow breathing suppression time, and a higher threshold volume needed to trigger the swallow response, all of which significantly raise the risk of aspiration [53].

Oral Manifestations of Obstructive Sleep Apnea (OSA)

For the oral cavity specifically, OSA drives mouth breathing as a compensatory airway strategy, which chronically reduces salivary flow, accelerates mucosal drying, and removes the buffering and antimicrobial protection that saliva provides, creating ideal conditions for caries progression, periodontal inflammation, and halitosis, effectively linking OSA back to nearly every oral health consequence discussed throughout this article [51].

The five domains discussed above illustrate the breadth of sleep deprivation's impact on oral health. Table 1 consolidates the key biological mechanisms, behavioral mediators, and downstream oral health manifestations discussed across these domains.

**Table 1:** Summary of the effects of sleep deprivation on oral health. CNS: central nervous system; MDA: malondialdehyde; SNS: sympathetic nervous system; TMD: temporomandibular disorders; IL-1: interleukin-1; IL-6: interleukin-6; IL-17: interleukin-17; TNF-α: tumor necrosis factor-alpha; SOD: superoxide dismutase; RAS: recurrent aphthous stomatitis; PYY: peptide YY.

Review domain	Specific sub-component/pathway	Key biological mechanisms and mediators	Clinical manifestations and oral outcomes
Immunological consequences	Periodontitis progression	SNS activation; elevated pro-inflammatory cytokines (IL-1, IL-6, IL-17, TNF-α); suppressed Th1 response [2,7,8].	Greater attachment loss, deeper probing depths, accelerated bone resorption, immune dysregulation [9].
	Mucosal healing	Elevated TNF-α, IL-1β, IL-6, IL-8 causing edema; increased oxidative stress (MDA, 8-OHdG); delayed SOD recovery [10,13,14].	Worsened inflammation, larger ulcers, delayed healing, higher risk of RAS [10].
Salivary environment and chemical shifts	Salivary hypofunction and xerostomia	Disrupted salivary circadian rhythm; reduced salivary volume and buffering capacity [18].	Xerostomia, morning hyposalivation, nocturnal awakenings, weaker mechanical rinsing defense [17].
	Dental caries and enamel vulnerability	Reduced salivary flow and sIgA; increased acidogenic microflora (*S. mutans*).	Faster plaque buildup, prolonged acid exposure, accelerated demineralization (caries).
	Halitosis	Reduced salivary flow combined with poor hygiene compliance.	Bacterial breakdown of debris, elevated volatile sulfur compounds, oral malodor.
Behavioral and lifestyle mediators	Glycemic dysregulation and diet	Reduced insulin sensitivity; altered appetite hormones (leptin, ghrelin, PYY).	Hyperinsulinemia, impaired glucose tolerance, increased caloric intake (~253-385 kcal/day), cravings for cariogenic carbohydrates.
	Tobacco use	Nicotine-circadian interaction; nighttime smoking fragments sleep; withdrawal-induced insomnia.	Greater nicotine dependence, higher cigarette use, relapse risk, worsened periodontal breakdown.
	Caffeine consumption	Adenosine receptor antagonism; reduced total sleep time (~45 min) and slow-wave sleep.	Delayed sleep onset, hypertensive response, IL-6 surge, compounding xerostomia and inflammation.
Neuromuscular and structural consequences	Traumatic dental injuries (TDIs)	Impaired motor coordination and alertness; daytime microsleeps; circadian misalignment.	More falls and collisions, raising risk of anterior dental trauma.
	Bruxism and TMD	Disrupted sleep architecture; heightened CNS arousal and stress.	More sleep bruxism, tooth wear, restorative failures, doubled TMD risk.
	Dysphagia and motor impairment	Impaired pharyngeal pressure and coordination; nocturnal reflux.	Higher dysphagia and aspiration risk, delayed food clearance, greater bacterial load.
Obstructive sleep apnea (OSA) comorbidity	Sleep-disordered breathing	Upper airway muscle hypotonia; blunted hypoxemia response; repeated airway collapse.	Oxygen desaturation, fragmented sleep, chronic mouth breathing driving xerostomia and oral pathology.
Awareness and clinical action	Patient self-monitoring and professional screening	High-sensitivity wearables (Oura, Fitbit, Apple Watch); validated STOP-Bang questionnaire.	Early detection via sleep tracking, dental screening of oral markers, referral to sleep specialists.

Awareness and action: Detecting and addressing sleep deprivation

The sections above have outlined how sleep deprivation can silently compromise nearly every aspect of oral health, from gum disease and tooth decay to jaw disorders and mucosal breakdown. Yet in clinical practice, sleep disorders remain significantly underdiagnosed, often because patients are unaware of their condition and because the signs are not always immediately linked to sleep in the minds of either the patient or the clinician. This section shifts the focus from understanding the problem to acting on it. It offers practical guidance for two key audiences: patients who wish to monitor and improve their own sleep, and dental professionals who are uniquely positioned to recognize the oral manifestations of sleep deprivation and refer patients toward appropriate care before systemic consequences take hold.

How Patients Can Monitor Their Sleep

Fortunately, patients do not need to wait for a clinical diagnosis to begin paying attention to their sleep. Several accessible tools and habits can help individuals gain meaningful insight into their sleep patterns and identify warning signs early.

Wearable Sleep Trackers

Consumer-grade wearable devices have made sleep monitoring increasingly accessible to the public. A 2024 study by Robbins et al. evaluated the accuracy of three widely used devices, the Oura Ring Gen3, Fitbit Sense 2, and Apple Watch Series 8, against polysomnography (PSG), which remains the clinical gold standard for sleep assessment. All three devices demonstrated a sensitivity of 95% or greater for distinguishing sleep from wakefulness. In terms of identifying specific sleep stages, the Oura Ring performed most consistently, showing no statistically significant difference from PSG in estimating wake, light sleep, deep sleep, or REM sleep. The Fitbit and Apple Watch showed moderate to substantial agreement with PSG but introduced some degree of overestimation or underestimation in certain sleep stages. While these devices are not diagnostic tools and should not replace clinical evaluation, they offer patients a practical and reasonably accurate means of tracking sleep duration and patterns over time, particularly for identifying trends such as consistently shortened sleep or frequent nighttime awakenings [54].

Sleep Diary and Behavioral Monitoring

For patients who prefer a low-technology approach, a sleep diary remains a clinically endorsed method of tracking sleep. Patients can record their bedtime, wake time, estimated total sleep duration, and subjective sleep quality on a nightly basis, with weekly review helping to identify problematic patterns. This approach requires no equipment and can surface issues such as irregular schedules or habitual short sleep that might otherwise go unnoticed [55,56].

Sleep Hygiene Practices

Alongside monitoring, adopting evidence-based sleep hygiene practices is essential. Current guidelines recommend that adults aim for seven to nine hours of sleep per night, maintain a consistent bedtime and wake time within a 30-minute window even on weekends, avoid screens for at least one hour before bed due to the alerting effects of blue light, and refrain from consuming caffeine after 2 PM. A cool, dark, and quiet sleep environment further supports sleep quality, and a relaxing pre-sleep routine of 30 to 60 minutes, such as reading or gentle stretching, has also shown benefit [55,56].

When to Seek Professional Help

Patients should be advised to consult a healthcare professional if they experience persistent difficulty sleeping for more than three months, significant daytime fatigue despite adequate time in bed, witnessed episodes of stopped breathing during sleep, or loud and chronic snoring. These signs may indicate an underlying sleep disorder such as obstructive sleep apnea, which requires formal clinical evaluation and cannot be adequately addressed through lifestyle adjustments alone [55,56].

How Dentists Can Detect Sleep Disorders

Routine dental visits provide a valuable opportunity for the early detection of sleep-related conditions. Unlike many healthcare settings, dentistry involves repeated, close-up examination of the oral cavity. Clinicians are thus able to observe a wide range of physical signs that may point toward underlying sleep disorders long before a formal diagnosis is made. A scoping review by Huang et al. confirmed that oral healthcare providers play a significant role in the screening, assessment, and management of several dental sleep-related conditions and stressed the importance of collaboration between dentists and physicians in ensuring patients receive appropriate care [57].

Oral Markers to Screen for Examinations

During routine examinations, dentists should be alert to a cluster of oral signs that, when present together, may suggest chronic sleep deprivation or an underlying sleep disorder. These include xerostomia (dry mouth), often caused by habitual mouth breathing during sleep; wear facets and grinding marks indicating sleep bruxism; and bleeding gums or signs of periodontitis, which can be exacerbated by the systemic inflammation associated with poor sleep. Additional signs include halitosis from reduced overnight salivary flow, an increased rate of carious lesions due to impaired salivary buffering capacity, and jaw pain or temporomandibular joint dysfunction. Burning mouth syndrome has also been identified as a dental sleep-related condition, given its established association with sleep disturbance. The presence of several of these findings in the same patient should prompt the clinician to consider a sleep-related etiology rather than treating each symptom in isolation [57].

Screening Tools

Beyond clinical observation, validated screening questionnaires represent a practical and time-efficient way for dentists to identify patients at risk of underlying sleep disorders. One of the most widely studied of these is the STOP-Bang questionnaire, which screens for eight key risk factors, including snoring, daytime tiredness, observed apnea, high blood pressure, body mass index, age, neck circumference, and gender. A study by Lonia et al. administered this questionnaire across three dental clinics and found that nearly half of patients screened at risk for OSA, with the majority who underwent further testing confirmed to have the condition. This suggests that a substantial proportion of dental patients may be carrying an undiagnosed sleep disorder, and the dental clinic may be the first to flag [58].

In addition to the STOP-Bang questionnaire, anatomical assessment during examination can further support risk stratification. Features such as a retrognathic mandible, a narrow or crowded upper airway, tongue scalloping, enlarged tonsils, and obesity are all recognized anatomical risk factors that can be noted during a standard oral examination. Where available, pulse oximetry can provide an additional layer of screening by detecting overnight oxygen desaturation patterns suggestive of disordered breathing during sleep [57].

The Dentist's Role: Screen, Refer, and Treat

It is important to clarify the boundaries of the dentist's role in this context. Oral healthcare providers are not responsible for diagnosing sleep disorders, as that remains within the domain of sleep medicine physicians. However, as this article has illustrated, the oral cavity accumulates evidence of sleep deprivation across multiple systems, from inflamed gums and eroded enamel to dry mouth, jaw dysfunction, and mucosal breakdown. The dentist is therefore uniquely positioned to connect these seemingly unrelated findings, raise the possibility of an underlying sleep disorder, and refer the patient to the appropriate specialist for formal evaluation. In cases where a diagnosis has already been established, such as mild to moderate OSA, dentists can also contribute directly to treatment through oral appliance therapy, a mandibular advancement device that repositions the jaw to maintain an open airway during sleep [57].

Limitations

This review has limitations that should be considered when interpreting these findings. Due to considerable heterogeneity in study designs, populations, and outcome measures across the 58 included articles, a narrative rather than a quantitative synthesis was conducted; this approach does not allow for pooled effect estimates or formal assessment of between-study heterogeneity. No formal risk-of-bias or quality assessment tool was applied to the individual included studies, which combine animal models, cross-sectional studies, randomized trials, and other reviews of varying methodological rigor. Exact search strings, screening numbers at each stage, and a PRISMA flow diagram were not reported, which limits the reproducibility of the search and selection process. Finally, much of the mechanistic evidence linking sleep deprivation to specific oral outcomes is derived from animal models or small human studies, and larger prospective human trials would help confirm causal directionality across the domains discussed. Incorporating standardized risk-of-bias assessment and more detailed search methodology in future work would further strengthen the evidence base in this area.

## Conclusions

Sleep is far more than nightly rest. As this review has demonstrated, it functions as a master regulator of oral health, operating through immunological, salivary, behavioral, neuromuscular, and structural pathways that together shape the condition of the oral cavity in ways that clinical practice has long underestimated. Insufficient or disrupted sleep amplifies pro-inflammatory cytokine activity, suppresses salivary defenses, destabilizes glucose metabolism, and impairs the neuromuscular coordination that swallowing and jaw function depend on. The oral consequences are wide-ranging: periodontal destruction, caries progression, mucosal breakdown, bruxism, temporomandibular dysfunction, dysphagia, and halitosis all emerge as downstream manifestations of a body chronically deprived of rest. What makes this particularly concerning is that many of these relationships are bidirectional, with conditions such as OSA and dysphagia actively worsening sleep quality in return, locking patients into cycles that are difficult to break without addressing both sides.

For the dental practitioner, this evidence points toward a concrete shift in how routine care is approached. The oral cavity quietly accumulates signs of sleep deprivation across multiple tissue systems, and the clinician who knows what to look for is well-placed to detect them early. Incorporating sleep-related screening into routine dental assessments, whether through validated questionnaires, anatomical risk evaluation, or recognition of characteristic oral markers, allows at-risk patients to be identified and referred before systemic consequences become harder to reverse. Integrating sleep health into dental practice is not a departure from the profession's scope; it is a natural and necessary step toward the kind of comprehensive, patient-centered care that modern evidence now demands.
